# Planning for ABF as part of reforming the Australian healthcare system

**DOI:** 10.1186/1472-6963-11-S1-A9

**Published:** 2011-10-19

**Authors:** L Fodero, J Scuteri, J Pearse, D Mazevska

**Affiliations:** 1 HealthConsult, Sydney, New South Wales, 2000, Australia; 2 Health Policy Analysis, St. Leonards, New South Wales, 1590, Australia

## Introduction

A key part of Australia’s national health reform policy is to implement activity-based funding (ABF) for public hospitals from 1st July 2012. The Council of Australian Governments (COAG) established the Health Reform Implementation Group (HRIG) to oversee the execution of the reforms. HRIG, in turn, created an ABF Sub Group to advise on implementing ABF.

The HRIG ABF Sub Group established five Advisory Working Groups (AWGs) aligned to the following workstreams: subacute, emergency, non-admitted, and mental health care. As well, the groups were aligned to the National Hospital Cost Data Collection (NHCDC). The terms of reference for the AWGs included “the development of a work plan for the implementation of ABF”, which will contribute to an “overall national work plan to implement ABF”.

## Methods

The HRIG ABF Sub Group decided that the proxy classifications systems to be used in the initial ABF implementation were Australian Refined-Diagnosis Related Groups (AR-DRGs) for acute admitted, Australian National Sub-Acute and Non-Acute Patient Classification System (AN-SNAP) for subacute, NHCDC Tier 2 Clinics for non-admitted, and Urgency Related Groups (URGs) for emergency services. Work Plans were needed to determine what was required to implement ABF by 1st July 2012 using the proxy classifications.

The planning methodology consisted of five parts. First, designing and distributing a survey for each workstream to assess the existing data collection and the availability of support infrastructure across states/territories. Second, preparing a scoping/gap analysis paper by analysing survey responses and interviewing state/territory representatives. Third, preparing the five draft Work Plans. Fourth, reviewing the draft Work Plans at the final meetings of the AWGs. Fifth, developing the Overarching Work Plan.

## Results

Across three meetings with the AWGs, Work Plans were developed which defined the projects that must be done to attain the minimum position in terms of ABF infrastructure in each workstream under the following headings: scope and data set coverage; classification; counting rules and unit of count; costing; funding model; support infrastructure; data quality assurance; and governance.

The planning process revealed the state-of-play across the ABF workstreams as:

• Subacute – The initial focus of AN-SNAP will need to be on activity occurring in designated services. Implementation will be easier to achieve for rehabilitation care and palliative care, where there is a relatively wide level of data collection. Considerable work is required to address issues for geriatric evaluation and management and psychogeriatric care.

• Non-admitted – The proxy classification is not yet completed, and the existing version is not used in any routine data collection. All the data available for NHCDC Tier 2 Clinics are produced by mapping from source data, which is collected using another classification system. This situation poses considerable challenges, particularly as there will be no historical data. and approaches to counting non-admitted patients vary greatly across states/territories.

• Emergency – Most states and territories have data collection systems that include the ‘Emergency Department (ED) diagnosis’, which is required to assign URGs. Initially, URGs can only be used in hospitals that have a recognised ED. UDGs (diagnosis not required) can be used for other emergency services. URGs will still need to be refined and updated prior to being used for ABF.

• Mental Health – The initial approach for mental health services is to use the proxy classification systems for the workstream into which the service falls. However, the best data on mental health services is in program-specific data collections, not in mainstream hospital systems. Also, there are significant limitations that need to be addressed in the classification systems in relation to mental health care.

• NHCDC – The challenge is to generate costs data to be used as an input to developing national cost weights and setting efficient prices. There is little consistency in the costing methodologies used by states/territories to generate the existing costs data. Extra studies undertaken to address this issue, and to add to the coverage of existing costs data, need to be finished by end of 2011. This poses sizeable challenges in terms of the capacity and capability of the available resources.

The Overarching Plan presents the minimum infrastructure needed to implement ABF within each workstream. It integrates the 99 projects identified across the five AWG Work Plans into 35 higher-level projects. It also identifies projects that are considered essential in order to enable national ABF implementation by the target date. In addition, a risk analysis is presented.

## Conclusions

The Work Plans show that it will be difficult to achieve a common starting point across states/territories for ABF by 1st July 2012. ABF readiness also varies across workstreams. Implementation strategies need to challenge states/territories to reach minimum position, but also be sufficiently flexible to allow for ABF to start even where the required data are not available.

## Disclaimer

The abstract does not necessarily represent the views of any Australian Government agency.

